# Clinical molecular testing for *ASXL1* c.1934dupG p.Gly646fs mutation in hematologic neoplasms in the NGS era

**DOI:** 10.1371/journal.pone.0204218

**Published:** 2018-09-17

**Authors:** Santiago Montes-Moreno, Mark J. Routbort, Elijah J. Lohman, Bedia A. Barkoh, Rashmi Kanagal-Shamanna, Carlos E. Bueso-Ramos, Rajesh R. Singh, L. Jeffrey Medeiros, Raja Luthra, Keyur P. Patel

**Affiliations:** 1 Department of Hematopathology, The University of Texas MD Anderson Cancer Center, Houston, TX, United States of America; 2 Pathology Department/Translational Hematopathology Lab, Hospital Universitario Marqués de Valdecilla/IDIVAL, Santander, Spain; University of Kansas Medical Center, UNITED STATES

## Abstract

*ASXL1* (additional sex combs like 1) is a gene that is mutated in a number of hematological neoplasms. The most common genetic alteration is c.1934dupG p.Gly646fs. Previous publications have shown that *ASXL1* mutations have a negative prognostic impact in patients with MDS and AML, however, controversy exists regarding the molecular testing of *ASXL1* c.1934dupG as polymerase splippage over the adjacent homopolymer could lead to a false-positive result. Here, we report the first study to systematically test different targeted next generation sequencing (NGS) approaches for this mutation in patients with hematologic neoplasms. In addition, we investigated the impact of proofreading capabilities of different DNA polymerases on *ASXL1* c.1934dupG somatic mutation using conventional Sanger sequencing, another common method for ASXL1 genotyping. Our results confirm that *ASXL1* c.1934dupG can be detected as a technical artifact, which can be overcome by the use of appropriate enzymes and library preparation methods. A systematic study of serial samples from 30 patients show that *ASXL1* c.1934dupG is a somatic mutation in haematological neoplasms including MDS, AML, MPN and MDS/MPN and often is associated with somatic mutations of *TET2*, *EZH2*, *IDH2*, *RUNX1*, *NRAS* and *DNMT3A*. The pattern of clonal evolution suggests that this *ASXL1* mutation might be an early mutational event that occurs in the principal clonal population and can serve as a clonal marker for persistent/relapsing disease.

## Introduction

*ASXL1* (additional sex combs like 1) is a gene that is mutated in a number of hematological neoplasms including Myelodysplastic Syndrome (MDS), Acute Myeloid Leukemia (AML), Myeloproliferative Neoplasms (MPN), Chronic Myelomonocytic Leukemia (CMML) and Chronic Myeloid Leukemia (CML). Different types of mutations occur including missense, nonsense and frameshift. The most common genetic alteration is a duplication of a guanine nucleotide at nucleotide position 1934 resulting in a frameshift and a premature stop codon that is 12 codons downstream of the insertion (referred to as ‘c.1934dupG; p.Gly646fs’). This particular insertion has been found in a substantial number of MDS and AML cases and accounts for most mutations in *ASXL1* according to COSMIC database and multiple case series so far reported [1–4]. Importantly this particular mutation is almost restricted to the hematopoietic lineage and previous publications have shown that *ASXL1* mutations have prognostic impact in patients with MDS [5] and AML [3, 6]. Thus, accurate genotyping of *ASXL1* c.1934dupG; p.Gly646fs mutations is clinically important.

Controversy exists, however, regarding the nature of *ASXL1* c.1934dupG mutation and some investigators have suggested that this mutation might not be a somatic mutation, but rather a germline polymorphism or artifact due to its location in an 8 base-pair mononucleotide guanine (G) nucleotide repeat[7]. Simple sequence repeats (SSR) in the genome have been shown to be prone to an increased rate of mutations via slipped strand mispairing[8, 9] and these mutations can occur both naturally and *in vitro* during enzymatic replication of SSRs. In this case, mutations are usually the result of insertion or deletion of repeats in the extending, or nascent, DNA strand sequence [10]. During PCR these artifacts occur at the greatest rate in homopolymer runs (mononucleotide repeats) of eight or more nucleotides [11] and importantly, their frequency can be reduced using fusion DNA polymerases [12].

To address this issue, we performed a systematic study of serial samples from 30 patients diagnosed with clonal hematological disorders using a multigene (28 gene) targeted approach and NGS sequencing using the Miseq platform. This study allowed us to produce additional data on the performance of different NGS protocols and an assessment of the optimal method for accurate genotyping in routine molecular diagnostics practice. Comparison between two NGS library preparation methods was performed. In addition we performed a validation study of the *ASXL1* c.1934dupG somatic mutation using conventional Sanger sequencing using two different proof reading DNA polymerases.

## Material and methods

### Patient selection

We retrieved all cases subjected to NGS analysis for the molecular diagnosis of haematological malignancies in the MD Anderson Cancer Canter’s Molecular Diagnostics Laboratory (MDL) database (≈ 6000 samples). A subset analysis included only patients with at least two different samples with *ASXL1* c.1934dupG (at least one sample should have a variant frequency (VF) >10%). 65 samples from 30 patients were identified. Clinical and pathological data were collected. All cases were classified according to the 2008 WHO classification. Clinical and laboratory data from standard of care routine clinical testing were obtained by electronic chart review. This study was approved by the Institutional Review Board of MD Anderson Cancer Center and performed in accordance with the Declaration of Helsinki. The need for informed consent was waived by the MD Anderson Cancer Center ethics committee for this retrospective chart review study. Subjects dataset included in the study was de-identified to protect patient privacy and anonymity.

### NGS using PCR amplicon-based and Haloplex probe capture-based library preparation methods

We prepared NGS sequencing libraries containing amplicons created by (i) PCR amplification of target regions (PA) using Truseq chemistry (Illumina) and (ii) Haloplex probe capture followed by PCR amplification of target regions (HCA) using Haloplex chemistry (Agilent). Both approaches targeted the coding regions of 28 genes implicated in myeloid neoplasms in DNA from fresh bone marrow aspirate samples using the MiSeq platform (Illumina, San Diego, CA). We used 250 ng of DNA to prepare the sequencing libraries. The genes included in this panel are: *ABL1*, *ASXL1*, *BRAF*, *DNMT3A*, *FGFR*, *EZH2*, *FLT3*, *GATA1*, *GATA2*, *HRAS*, *IDH1*, *IDH2*, *IKZF2*, *JAK2*, *KIT*, *KRAS*, *MDM2*, *MLL/KMT2A*, *MPL*, *MYD88*, *NOTCH1*, *NPM1*, *NRAS*, *PTPN11*, *RUNX1*, *TET2*, *TP53*, and *WT1*. Other genes relevant for haematolymphoid neoplasms such as *CEBPA* are routinely screened using a stand-alone test based on Sanger sequencing. Following successful library generation and purification, equal quantities of DNA from each sample were used for multiplex paired-end sequencing on the MiSeq personal genome sequencer using the MiSeq Reagent Kit v2 (500 cycles). Human genome build 19 (hg19) was used as the reference for sequence alignment. Successful sequencing was indicated by a Q30 score of more than 85%. MiSeq Reporter and Agilent SureCall were used for variant calling for Truseq and Haloplex workflow respectively. To rule out the impact of NGS informatics pipeline on the differential frequency of *ASXL1* mutation calls between the two library preparation methods, we ensured that both the pipelines had a VAF cut-off of 1% for calling these mutations. An in-house developed post-variant calling statistical curation tool, Oncoseek, was used for filtering vcf calls and the Integrative Genomics Viewer (IGV) was used for visualization. Variants with more than 5% allelic frequency with a minimum coverage of 250X in both directions were considered. Variant frequency, together with data from the COSMIC, dbSNP (137 and 138) and 1000 genome project databases were used to infer the somatic nature of the changes.

### Sanger sequencing of *ASXL1*

Bidirectional Sanger sequencing was used to confirm the presence of frameshift mutations in codon 646 (c.1934dupG; p.Gly646fs) in exon 12 of *ASXL1*. DNA extracted from fresh bone marrow aspirate samples was subjected to PCR using two different DNA polymerases, Phusion high/fidelity DNA polymerase (ThermoFisher) and KAPA HiFi Ready Mix (KAPA BIOSYSTEMS). 2μL of DNA at a concentration of 5 ng/μL were used for testing. PCR primer sequences were GGACCCTCGCAGACATTAAA and CACCACCATCACCACTGCT. M13-tags attached to PCR primers were utilized for cycle sequencing. The 3130xl Genetic Analyzer was used for sequencing. A negative cell line (HL60) was used as control. Seqscape software v2.7 and Chromas 2.4.4 were used for electropherogram visualization.

## Results

### *ASXL1* c.1934dupG is a somatic mutation when found in a significant proportion of the clonal population by NGS. Low Variant Allele Frequency is related with in vitro enzymatic replication of SSRs

The first evidence of the impact of NGS library preparation method on ASXL1 p.C1934dupG detection came from a significantly higher number of *ASXL1* c.1934dupG mutations called at >1% variant allele frequency (VAF) in the PCR-only amplification (PA) analysis cohort (2870/2991; 96%) compared to the Haloplex probe capture followed by PCR amplification (HCA) analysis cohort (250/3071; 8%) (p value <0.0001).

**Fig 1:**
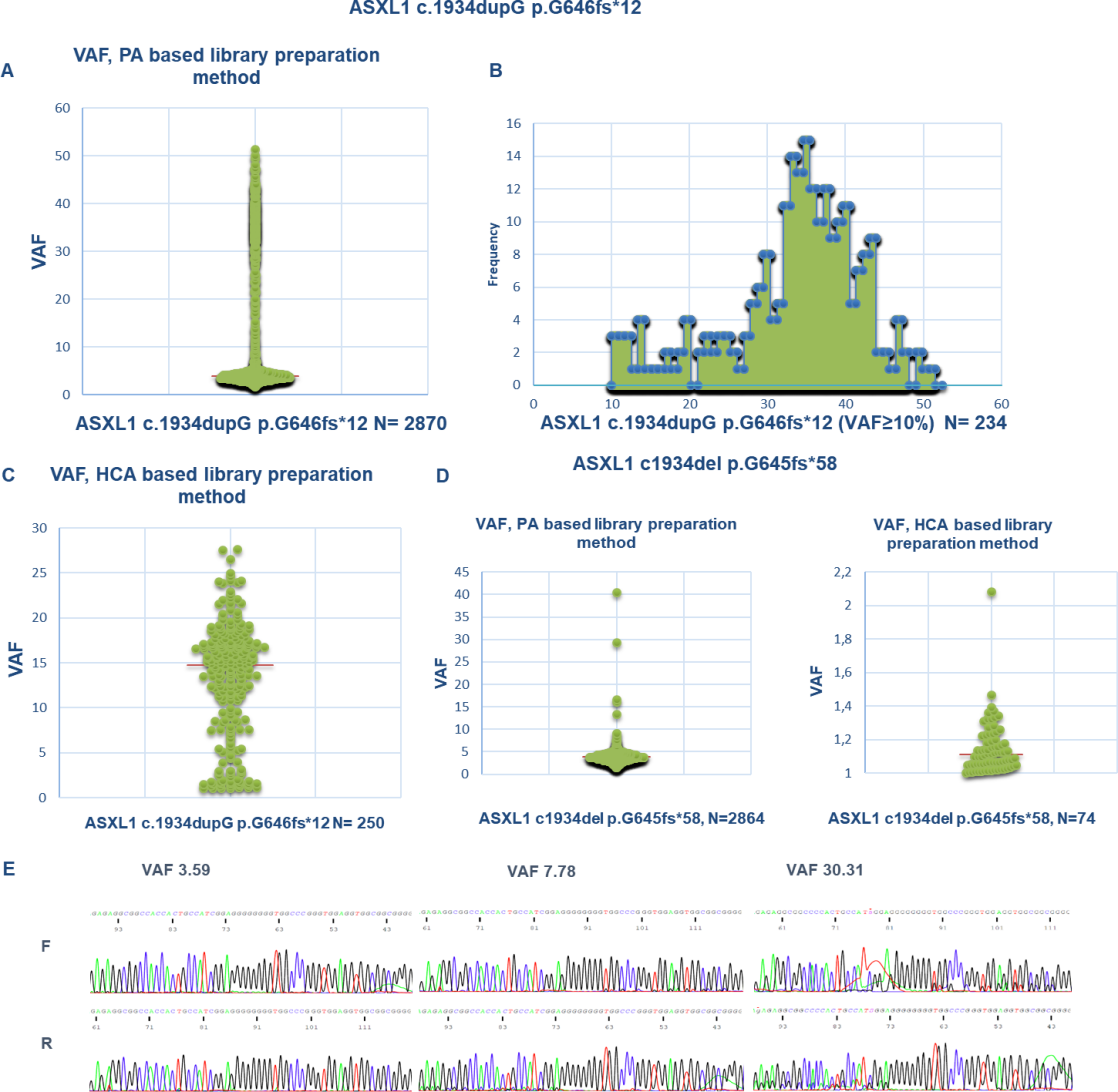
**A**. VAF scatterplot plotting all the clinical samples studied by NGS with direct PCR amplification (PA) based library preparation chemistry found to harbor ASXL1 c.1934dupG (n = 2870). Majority of the cases (2636, 92%) have a variant frequency below 10%. Y axis: VAF. **B**. 234 samples (8%) were identified with *ASXL1* c.1934dupG with VAF≥10%. The maximum VAF observed in the series was 51.4. X axis: VAF, Y axis: number of samples. **C**. VAF scatterplot showing all the clinical samples studied by HCA-based NGS library preparation chemistry found to harbor *ASXL1* c.1934dupG (n = 250).Y axis: VAF **D**. Left panel: VAF scatterplot showing all the clinical samples studied by NGS with amplicon based library preparation chemistry found to harbor *ASXL1* c.1934del p.G645fs (n = 2864). As depicted, most of the cases (99%) have a variant frequency below 10%. The maximum VAF observed in the series was 40.36. Y axis: VAF. Right panel: VAF scatterplot showing all the clinical samples studied by HCA-based NGS found to harbor *ASXL1* c.1934del p.G645fs (n = 74). Y axis: VAF. **E**. Three electropherograms of cases with *ASXL1* c.1934dupG quantified as 3.59, 7.78 and 30.31 respectively by NGS after amplicon based library preparation. As shown, no minor clonal sequence is found in the case with a VAF<5%.

VAF scatterplots for *ASXL1* c.1934dupG mutation in the AB chemistry (n = 2870) showed that the vast majority of the mutation calls (2636, 92%) had a VAF of <10%. Only 234 (8%) samples were identified with *ASXL1* c.1934dupG with VAF≥10% (Fig 1). Interestingly a similar number (n = 2864) of *ASXL1* c.1934del p.G645fs was found (99.99% of the observations were at a VAF below 10%) with comparable VAF. These data suggest that both *ASXL1* c.1934dupG and *ASXL1* c.1934del p.G645fs are frequently found when PA is used for library preparation, accounting for a very limited percentage of the fraction of reads (usually ≤5% of the VAF), likely representing artifacts of library preparation and/or sequencing. In comparison, the HCA library preparation method showed much lower rate of ASXL1 p.1934dup (n = 250/3071, 8%) and ASXL1 p.1934del (n = 74/3071, 2%) mutation calls. In addition to the lower overall population frequency for *ASXL1* c.1934dupG, the cases using HCA methods showed higher median VAF (14.75, range 1–27, n = 250) compared to the lower median VAF for cases using PA library preparation methods (3.87, range 1.73–51.4, n = 2870) (p-value (U Mann-Whitney test) <0.0001), suggesting that the artifact occurs during library preparation step.

### Reduced rate of *ASXL1* c.1934dupG after the use of Phusion DNA polymerases

Since the rate of mutations due to slipped strand mispairing has been shown to be reduced by the use of Phusion high fidelity DNA polymerase[12], Sanger sequencing was performed on exon 12 of the *ASXL1* gene to detect *ASXL1* c.1934dupG using two different DNA polymerases in the first PCR reaction, Phusion high/fidelity DNA polymerase and KAPPA HiFi Ready Mix. 19 cases were used for the comparison of the presence of stutter products in sequence chromatograms. The presence of a minor sequence showing *ASXL1* c.1934dupG is reduced in cases after PCR amplification using Phusion high/fidelity DNA polymerase. This difference was obvious for cases with a VF <5% as quantified by NGS. None of these 14 cases showed stutter products after using Phusion high/fidelity DNA polymerase. In contrast, 5 cases with VF > 10% showed a minor sequence with *ASXL1* c.1934dupG in a sub-hemyzigous fraction (Fig 1E and S1 Fig).

Collectively, these data suggest that both *ASXL1* c.1934dupG and ASXL1 c.1934del p.G645fs, when quantified at low levels (<5% by NGS), are likely an artifact induced during the PCR reaction for library preparation. A borderline area between 5% and 10% VF exists and caution is required in the diagnostic interpretation of these cases. Previous evidence of *ASXL1* c.1934dupG at confidence levels (VF>10%) should be required to report a positive call when VAF is low.

### *ASXL1* c.1934dupG is a somatic mutation when found in a significant proportion of the clonal population

**Fig 2:**
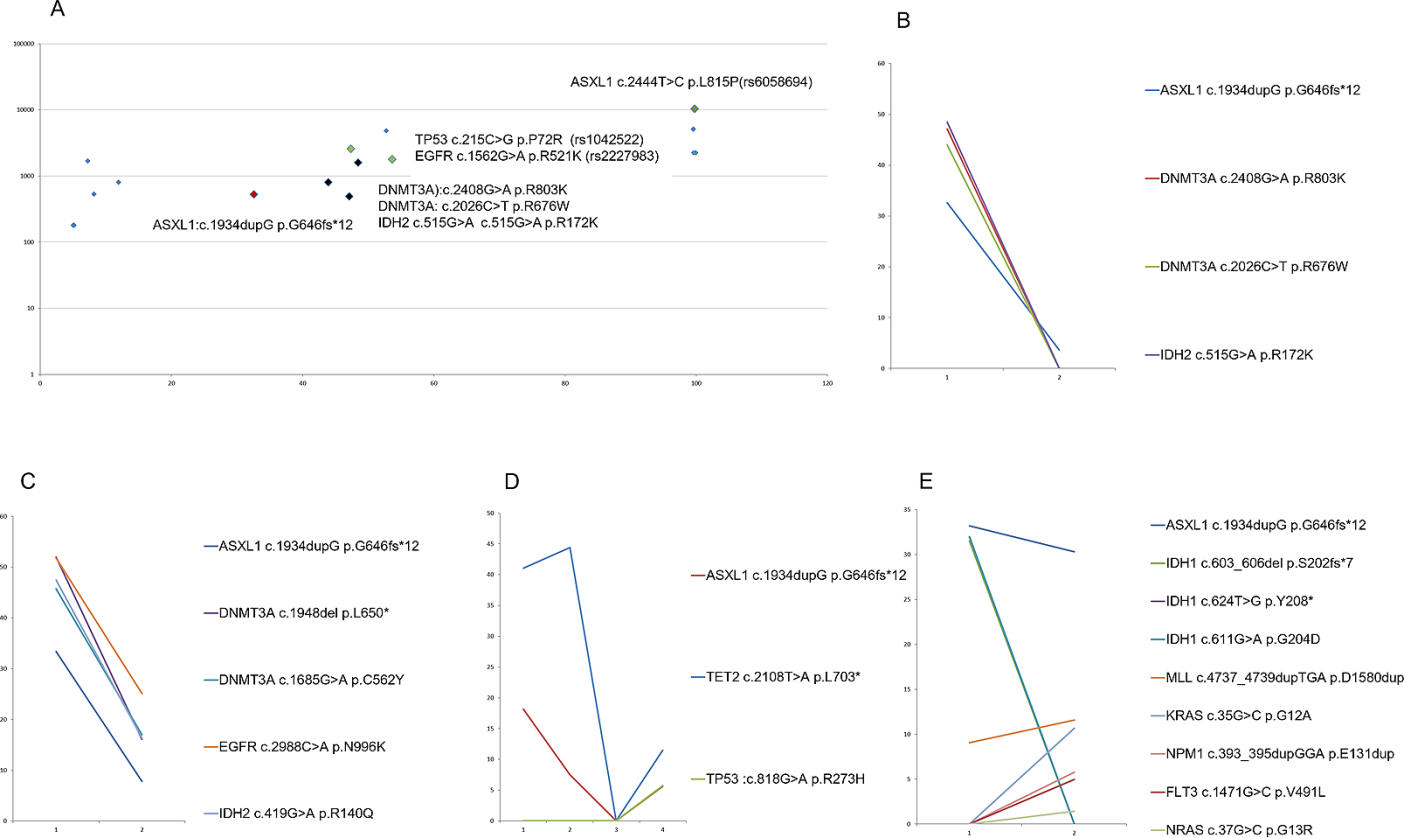
**A**. Variant allele frequency (X axis) was plotted against variant coverage (log10 scale, Y axis) in patient n2. *ASXL1* c.1934dupG was present at a VAF different than those found for well recognized SNPs (*TP53* c215 C>G, *EGFR* c1562 G>A and *ASXL1* c2444 T>C). **B, C, D and E**. VAF (Y axis) is plotted in two different time points (X axis) in four different patient samples.**B**. Patient n2 (male, 65 year old) samples are shown. First sample (MDS RAEB-2, 15% blasts in BM aspirate count) was obtained prior to therapy. A second 28-gene NGS analysis was done 20 months after alo-SCT (trilineage haematopoiesis, 1% blast count in BM aspirate). A dramatic reduction in VAF for all the mutations found at diagnosis (*ASXL1* c.1934dupG, *DNMT3A* c.2408G>A, *DNMT3A* c.2026 C>T and *IDH2* c515G>A) is evident after SCT. **C.** Patient n6 (female, 62 year old) was diagnosis as AML with MDS related changes (52% blasts in BM aspirate count)). The patient underwent haploidentical SCT. On day #76 after transplant the patient relapsed (29% blasts in BM aspirate count). As shown, both samples share the same mutational profile, characterized by *ASXL1* c.1934dupG, *DNMT3A* c.1948del, *DNMT3A* c.1685G>A, *EGFR* c.2988C>A and *IDH2* c.419G>A. **D.** Patient n30 (male, 64 year old) samples are shown. First and second samples were diagnosed as MPN (*JAK2* negative post ET-myelofibrosis, fibrosis grade 3 and osteosclerosis, blast count in BM aspirate was 1% and 8%). The third sample was obtained 30 days after allogeneic SCT. Pathological diagnosis was hypocellular BM (5% blasts) with osteosclerosis. The fourth sample was obtained 7 months after SCT and the pathological diagnosis was cellular bone marrow with fibrosis grade 2. A decline in the donor chimerism both in the myeloid and T-lineage cell types was observed at that time point. As shown in the figure, a dramatic reduction of both *ASXL1* c.1934dupG and *TET2* c.2108T>A p.1703* is seen after allogeneic SCT, in correlation with BM cellularity. Both *ASXL1*, *TET2* and a new clonal population characterized by *TP53* c.818G>A p.R273H mutation arises after therapy. **E.** Patient n1 (male, 79 year old) samples are shown. First sample was diagnosed as MDS/MPN (7% blast count). After 16 cycles of Vidaza/Revlimid the patient showed persistent MDS/MPN with 17% blast count. As shown in the figure, there is a shift in the clonal composition of the disease with a decrease in the three *IDH1* mutations first identified (*IDH1* c603_606del p.S202fs*7, *IDH1* c624T>G p.Y208* and *IDH1* c611G>A p.G204D) after therapy, but the emergence of new clones characterized by *FLT3* c.1471G>C p.V491L, *NPM1* c393_395dupGGA p. E131dup, *KRAS* c35G>C p.G12A and *NRAS* c.37G>C p.G13R. Both *ASXL1* c.1934dupG p.G646fs*11 and *MLL/KMT2A* c4737_4739dupTGA p.D1580dup remain relatively constant along time.

After establishing the optimal assay conditions and interpretation cutoff for ASXL1 c.1934dupG mutation detection, we sought to understand the somatic versus germline origin of this mutation. 59 of 65 serial samples from 30 patients had at least 1 positive result for *ASXL1* c.1934dupG (considering VF ≥10% as a threshold). This determination is especially important in the clinical setting as control germline samples are not routinely tested. First, we compared the VAF of c.1934dupG p.Gly646fs to the VAF found for well recognized SNPs identified in the samples. In the case of SNPs, VAF is constant, ≈50 or ≈100% whereas for c.1934dupG; p.Gly646fs the VAF was variable in different samples from the same patient at different time-points (Fig 2).

Additional evidence for the somatic origin of *ASXL1* mutation was its VAF correlation with other somatic mutations found in serial samples from the same patient (Fig 2) indicating a somatic origin in the clonal cells. In 28 of 30 (93%) patients *ASXL1* c.1934dupG mutation was found in association with another mutation included in the multigene panel. Changes in VAF of the somatic mutations identified at different time-points in a single patient, including *ASXL1* c.1934dupG, suggesting that ASXL1 c.1934dupG burden evolved in concert with other driver mutations found in these samples (see later).

### *ASXL1* c.1934dupG correlates with other major mutated driver genes

*ASXL1* c.1934dupG mutation as a single somatic mutation found in only two of 30 (6%) cases. In the majority of the cases (94%) the mutation was found in combination with other somatic mutations (median 4 somatic mutations per case, range 1–8). A detailed overview of the mutational profile of these cases is found in S2 Fig.

Only two cases (n11 and n14), with a diagnosis of MPN (Myelofibrosis post Essential Thrombocytemia) and AML, had a single mutation in *ASXL1*, without mutations in the other genes tested. In addition, two cases had at least one additional mutation in *ASXL1*, together with *ASXL1* c.1934dupG. One case (n12) had a missense point mutation (*ASXL1*:c.3151C>T p.R1051C) and the second case (n10) had two additional frameshift mutations leading to stop codons (*ASXL1*:c.2139del p.M713fs*12 and *ASXL1*:c.2423dupC p.A809fs*12).

*ASXL1* c.1934dupG was found co-mutated with *TET2* (14 events), *EZH2* (11 events), *IDH2* (10 events), *RUNX1* (10 events), *NRAS* (7 events) and *DNMT3A* (6 events). The number of events was <5 for *IDH1*, *MLL/KMT2A*, *PTPN11*, *KRAS*, *FLT3*, *JAK2*, *MPL*, *KIT*, *GATA2*, *TP53* and *NOTCH1*.

*DNMT3A*, *IDH1*, *IDH2* and *RUNX1* mutations were frequent in MDS, MDS/MPN and AML NOS, AML with MDS related changes and AML secondary to MPN. *FLT3* and NPM1 mutations were restricted to cases with AML NOS and MDS/MPN (*FLT3*:c.2134A>T p.R712W, *FLT3*:c.2812_2820dupAATTTGACT p.N938_T940dupNLT, *FLT3*:c.2503G>T p.D835Y, NPM1: c.393_395dupGGA p.E131dup). All mutations (*DNMT3A*, *IDH1*, *IDH2*, *RUNX1*, NPM1 and *FLT3*) were absent in all 5 patients with MPN. MPN patient samples were characterized by the presence of *JAK2* (c.1849G>T p.V617F), *KIT* (c.1655T>C p.M552T) and *MPL* mutations (*MPL*:c.1544G>T p.W515L and *MPL*:c.1841G>A p.G614E). Of these genes, other *MPL* mutations were found in one additional case with a diagnosis of AML NOS (*MPL*:c.1277G>A p.R426Q).

Next, we evaluated clonal dynamics in these 30 ASXL1 c.1934dupG cases. A clonal evolution pattern was noted in 46% of the patients. Most cases were characterized by an expansion of a minor clone carrying the vast majority of the primary tumor mutations that survived and expanded at progression/relapse. In a minority of patients (10%) we identified a pattern consistent with acquisition of progression/relapse specific mutations in *FLT3*, *NPM1*, *KRAS*, *NRAS*, *MPL*, *NOTCH1* and *TP53*.

## Discussion

*ASXL1*, located on chromosomal region 20q11, is mutated in ≈20% of patients with MDS, ≈35% of MPN, ≈30% of secondary AML, ≈7% of the *de novo* AML and up to 40% of patients with MDS/MPN[2, 13]. There is evidence showing a consistent association between *ASXL1* mutations and adverse survival in patients with MDS and AML NOS[3, 5, 13–15]. Different types of mutations in *ASXL1* have been described, with most being nonsense mutations and insertions/deletions. Most *ASXL1* mutations are located in the 5′ region of the last exon (exon 12), c.1934dupG; p.Gly646fs being the most common [4, 13, 16]. This mutation is almost restricted to the haematopoietic lineage with the notable exception of MSI-high colorectal cancer cell lines where *ASXL1* mutations have been associated with microsatellite instability and predicted NMD (nonsense mediated decay) resistant derived neoantigens [17].

This particular mutation, c.1934dupG; p.Gly646fs, however, has been the subject of controversy. It has been suggested that this mutation represents an artifact due to *in vitro* slipped strand mispairing during PCR amplification, or is even not somatic in nature, but germline[7]. Here we compared NGS and Sanger capillary electrophoresis for the detection of the mutation and used different methods for PCR amplification and library preparation. We show that c.1934dupG; p.Gly646fs, when found at low VAF, can result from low fidelity DNA replication during the library preparation step for NGS. The use of Phusion high/fidelity DNA polymerase reduced the rate of sequences containing an artifactual c.1934dupG as detected by Sanger sequencing. These data are consistent with previously published data on the improvement of quality of DNA amplification in sequences with a high content of simple sequence repeats [11, 12] by the use of Phusion high/fidelity DNA polymerases. It is apparent that the use of different types of polymerases is a variable that critically affects the quality of the amplification of that particular type of DNA region. A likely explanation for the superiority of Phusion high/fidelity DNA polymerases is that the presence of an additional DNA binding domain (afforded by the addition of Sso7d protein) effectively increases the contact surface with the DNA, enabling accurate replication of larger repeat regions, as has been suggested[12]. Importantly, we show that VAF quantification provided by the NGS analysis pipeline is helpful for distinguishing artifactual c.1934dupG from biological c.1934dupG mutations. From the molecular diagnostic standpoint, we suggest a conservative cutoff of 10% to consider a c.1934dupG mutation true when NGS experiments are performed using amplicon-based library preparation platforms, based on two observations: first, samples with a VAF ≥ 10% for *ASXL1* c.1934dupG mutation were confirmed using Sanger sequencing with Phusion high/fidelity DNA polymerase (Fig 1 and S1 Fig); second, the population based scatter plot comparing VAF in every NGS experiment using amplicon-based library preparation from the clinical samples cohort in our center (Fig 1) showed a dramatic reduction in the frequency of cases with a VAF above 10. Caution is required in the diagnostic interpretation of those cases and earlier evidence of a *ASXL1* c.1934dupG at confidence levels (VF>10%) should be required to report a low-level positive call. Otherwise a statement of the subclonal nature of the mutation and undetermined clinical significance for those cases within the borderline range (5–10% VF) is required. In PA-based sequencing, the initial extension and ligation step using genomic DNA as the template may give rise to targets with artifacts that get propagated at the PCR amplification step. In comparison, the use of DNA fragments captured directly from the enzymatically-digested genomic DNA in HCA method likely minimizes the rate of artifacts in the targets prior to PCR amplification.

Here we provide convincing evidence of the somatic nature of c.1934dupG; p.Gly646fs based on an analysis of the clonal allelic frequency and clonal evolution patterns in a series of 30 patients with serial samples analyzed by NGS. First, we compared the VAF of c.1934dupG; p.Gly646fs to the VAF found for well recognized SNPs identified in the samples characterized by a constant VAF in serial samples. In the case of SNPs, VAF is ≈50 or ≈100% whereas for c.1934dupG; p.Gly646fs the VAF was variable in different samples from the same patient and the trend (increase or decrease) at different timepoints correlated well with the VAF of other somatic mutations found in the same samples. These findings are consistent with studies showing acquired nature of ASXL1 mutations and their correlation with myeloid transformation[18]. In addition, Metzeler et al also showed presence of ASXL1 mutation in leukemic cells and not in germline control samples[14].

*ASXL1* c.1934dupG; p.Gly646fs was found in diverse groups of myeloid neoplasms (MDS, MDS/MPN, AML and MPN) in this study and across all age groups (median, 70 years; range 54–85 years). There was a striking male to female ratio of 3:1 and 60% of the patients were dead in the last follow up available. Since selection of the cases was based on the positivity in at least one sample for *ASXL1* c.1934dupG mutation and the availability of serial samples from the same patient, we believe this series likely reflects a random selection of myeloid neoplasms with *ASXL1* c.1934dupG mutation.

*ASXL1* c.1934dupG mutation as a single somatic mutation found in only two of 30 (6%) cases. In other cases the mutation was found in combination with other somatic mutations included in the targeted sequencing panel used (median 4 somatic mutations per case, range 1–8). In two cases, more than one mutation in exon 12 of *ASXL1* were identified, including missense single nucleotide substitutions, single nucleotide substitutions and frameshift insertions and deletions. As previously described, *ASXL1* mutations are frameshift and nonsense mutations. These mutations are usually hemizygous[13] (of note all cases in this study had a VAF below 50% for *ASXL1* c.1934dupG). Interestingly previously published data suggest that *ASXL1* mutations require the co-occurrence of other oncogenic gene mutations to lead to hematopoietic transformation [19]. Our results *in vivo*, showing *ASXL1* mutations with other gene mutations in 94% of myeloid neoplasms assessed, support this notion.

*ASXL1* mutations were found, in diverse types of myeloid neoplasms and, in most cases in combination with other gene mutations. The most commonly co-mutated genes were *TET2*, *EZH2*, *IDH2*, *RUNX1*, *NRAS and DNMT3A*. These data are consistent with previously published data suggesting that *TET2*, *ASXL1*, *DNMT3A* are early initiation mutations in the development of clonal hematopoietic expansion[20–22].

The spectrum of disease entities found to harbor *ASXL1* mutations is consistent with previously reported findings. We identified *ASXL1* c.1934dupG in four cases of MPN (1 case of primary myelofibrosis and 3 cases diagnosed as ET with secondary fibrosis). None of our cases was diagnosed as PV in which *ASXL1* mutations are rare[13]. Seven of cases were diagnosed as MDS/MPN, and five of these fulfilled criteria for CMML, a disease in which *ASXL1* mutations are frequent, ≈45%[2, 13]. 13 patients were diagnosed as AML, in 1 case after a diagnosis of CMML, in 4 cases after MPN, in 3 cases with MDS related changes and in 5 cases as *de novo* AML. *ASXL1* mutations have been found more frequently in cases of secondary AML (≈30%) in comparison with *de novo* cases (≈6.5%) and characteristically in AML with MDS-related changes[13]. Cases of AML with MDS related changes are characterized, at the molecular level, by a high frequency of *ASXL1* mutations and a low rate of *NPM1*, *FLT3* and *DNMT3A* mutations[23]. Here we identified 3 cases; none had *FLT3* ITD or point mutations, nor *NPM1* mutations and only one out of three of these cases had *DNMT3A* mutations (case n6, *DNMT3A*:c.1685G>A p.C562Y, *DNMT3A*:c.1948del p.L650*). Regarding secondary AML from previous MPN disease, all four cases with *ASXL1* mutation lacked *NPM1* mutations and were characterized by a coexistence of *ASXL*1 and *TET2*, *EZH2* and *JAK2* mutations. These data are in agreement with previous knowledge on AML secondary to MPN, suggesting a unique molecular route involving *ASXL1* mutations as early drivers of the disease [24]. Six cases were diagnosed as MDS. Of these, co-mutated genes with *ASXL1* were *EZH2*, *RUNX1*, *TET2*, *IDH2* and *TP53* (see S2 Fig). *TP53*, *EZH2*, *RUNX1*, and *ASXL1* mutations have been found to be independent predictors of survival in MDS patients, in multivariate models with International Prognostic Scoring System risk category (IPSS)[25]. Interestingly a relationship between *ASXL1* mutations and MDS pathogenesis has been found since conditional *ASXL1* gene knock-out in a murine model developed a phenotype consistent with MDS with myeloproliferative features[26].

Regarding clonal dynamics, most cases were characterized by an expansion of a minor clone carrying the vast majority of the primary tumor mutations that survived and expanded at progression/relapse. In a minority of patients (10%) we identified a pattern consistent with acquisition of progression/relapse specific mutations in *FLT3*, *NPM1*, *KRAS*, *NRAS*, *MPL*, *NOTCH1* and *TP53* [27].

Our results may have direct implications in the design of methods to quantify minimal residual disease based on this ASXL1 hotspot mutation using NGS. Recent data suggest that NGS sequencing methods can be used to track neoplastic clones with comparable sensitivity to qPCR based methods and provide relevant prognostic information, as is the case for RUNX1 mutant alleles[28]. In the case of *ASXL1* c.1934dupG, our results using amplicon based targeted NGS show important limitations for MRD monitoring, due to the presence of a potential high rate of false positive results in cases with a VAF below 10%. Haloplex probe capture-based library preparation methods might be a better approach to detect low-level *ASXL1* c.1934dupG, combined with deep sequencing to ensure robust detection of the mutation. The relatively high frequency and clinical impact of *ASXL1* c.1934dupG mutation in MPN, CMML, MDS/AML cases makes *ASXL1* a good candidate for disease monitoring using ultrasensitive detection by NGS that could be applied to DNA derived from bone marrow or plasma (cftDNA)[29].

In summary, our study provides a definitive proof that *ASXL1* mutation detection in clinical setting can be affected by proofreading activity of DNA polymerase as well as NGS library preparation methods. We also confirmed that *ASXL1* c.1934dupG is a somatic mutation that is usually clonally related and associated with somatic mutations in *TET2*, *EZH2*, *IDH2*, *RUNX1*, *NRA*S and *DNMT3A*. The pattern of clonal evolution suggests that this particular mutation might be an early mutational event occurring in the principal clonal population and can serve as a clonal marker for persistent/relapsing disease.

## Supporting information

S1 Fig Electropherograms of cases with *ASXL1* c.1934dupG quantified at very low level (<5% by NGS, cases s1 to s14) or confidence levels (>10%, cases s15 to s19). Column A represents samples sequenced after the use of Phusion high/fidelity DNA polymerase and column B represents the same samples sequenced after PCR amplification using KAPPA HiFi Ready Mix. As depicted the presence of a minor sequence showing *ASXL1* c.1934dupG in cases with low VF is reduced in cases after PCR amplification using Phusion high/fidelity DNA polymerase (column A).
(PDF)

S2 Fig Graphical representation of mutational profile along the series of 30 patients. Each column represents a single patient. Information regarding diagnosis, age, gender, outcome in the last follow-up, total number of mutations and specific mutations found is given. *ASXL1* c.1934dupG was found co-mutated with *TET2* (14 events), *EZH2* (11 events), *IDH2* (10 events), *RUNX1* (10 events), *NRAS* (7 events) and *DNMT3A* (6 events). The number of events was <5 for *IDH1*, *MLL/KMT2A*, *PTPN11*, *KRAS*, *FLT3*, *EGFR*, *JAK2*, *MPL*, *KIT*, *GATA2*, *TP53 and NOTCH1*. *DNMT3A*, *IDH1*, *IDH2* and RUNX1 mutations were frequent in MDS, MDS/MPN and AML NOS, with MDS related changes and AML secondary to MPN. *FLT3* and *NPM1* mutations were restricted to cases with AML, NOS and MDS/MPN (FLT3:c.2134A>T p.R712W, FLT3:c.2812_2820dupAATTTGACT p.N938_T940dupNLT, FLT3:c.2503G>T p.D835Y, NPM1: c.393_395dupGGA p.E131dup). All these mutations (*DNMT3A*, *IDH1*, *IDH2*, *RUNX1*, *NPM1* and *FLT3*) were however absent in all 4 patients with MPN. MPN patient samples were characterized by the presence of JAK2 (c.1849G>T p.V617F), KIT (c.1655T>C p.M552T) and MPL mutations (MPL:c.1544G>T p.W515L and MPL:c.1841G>A p.G614E). Of these genes, other MPL mutations were found in one additional case with a diagnosis of AML NOS.
(PDF)
