# Supplementary material for: Clinical molecular testing for ASXL1 c.1934dupG p.Gly646fs mutation in hematologic neoplasms in the NGS era
Source: PLoS One. 2018 Sep 17;13(9):e0204218. doi: 10.1371/journal.pone.0204218 (PMC6141087; doi:10.1371/journal.pone.0204218)
Supplement: S1 Fig — Column A represents samples sequenced after the use of Phusion high/fidelity DNA polymerase and column B represents the same samples sequenced after PCR amplification using KAPPA HiFi Ready Mix. As depicted the presence of a minor sequence showing ASXL1 c.1934dupG in cases with low VF is reduced in cases after PCR amplification using Phusion high/fidelity DNA polymerase (column A). (PDF) [file pone.0204218.s001.pdf]

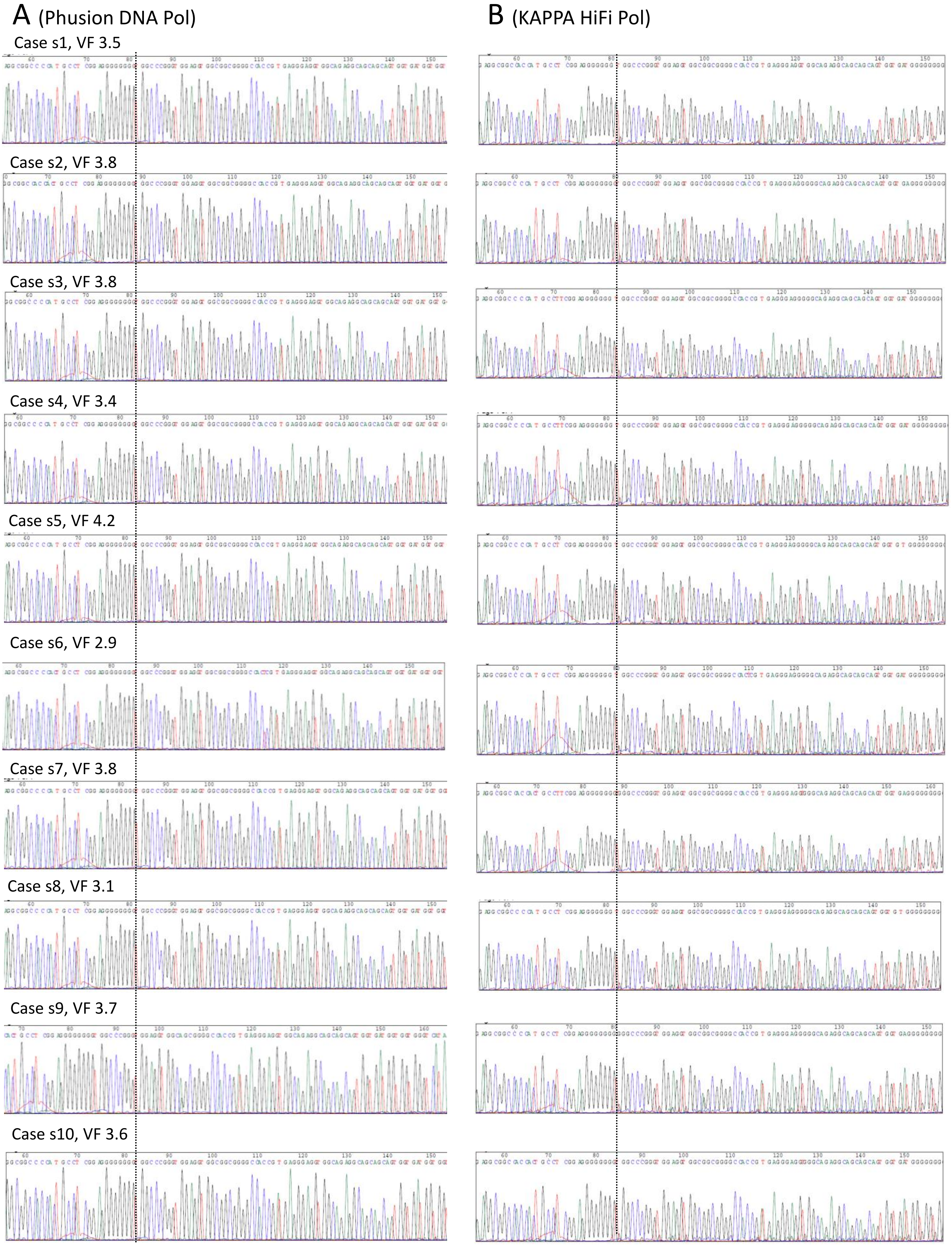
A (Phusion DNA Pol)
B (KAPPA HiFi Pol)
Case s1, VF 3.5
Case s2, VF 3.8
Case s3, VF 3.8
Case s4, VF 3.4
Case s5, VF 4.2
Case s6, VF 2.9
Case s7, VF 3.8
Case s8, VF 3.1
Case s9, VF 3.7
Case s10, VF 3.6

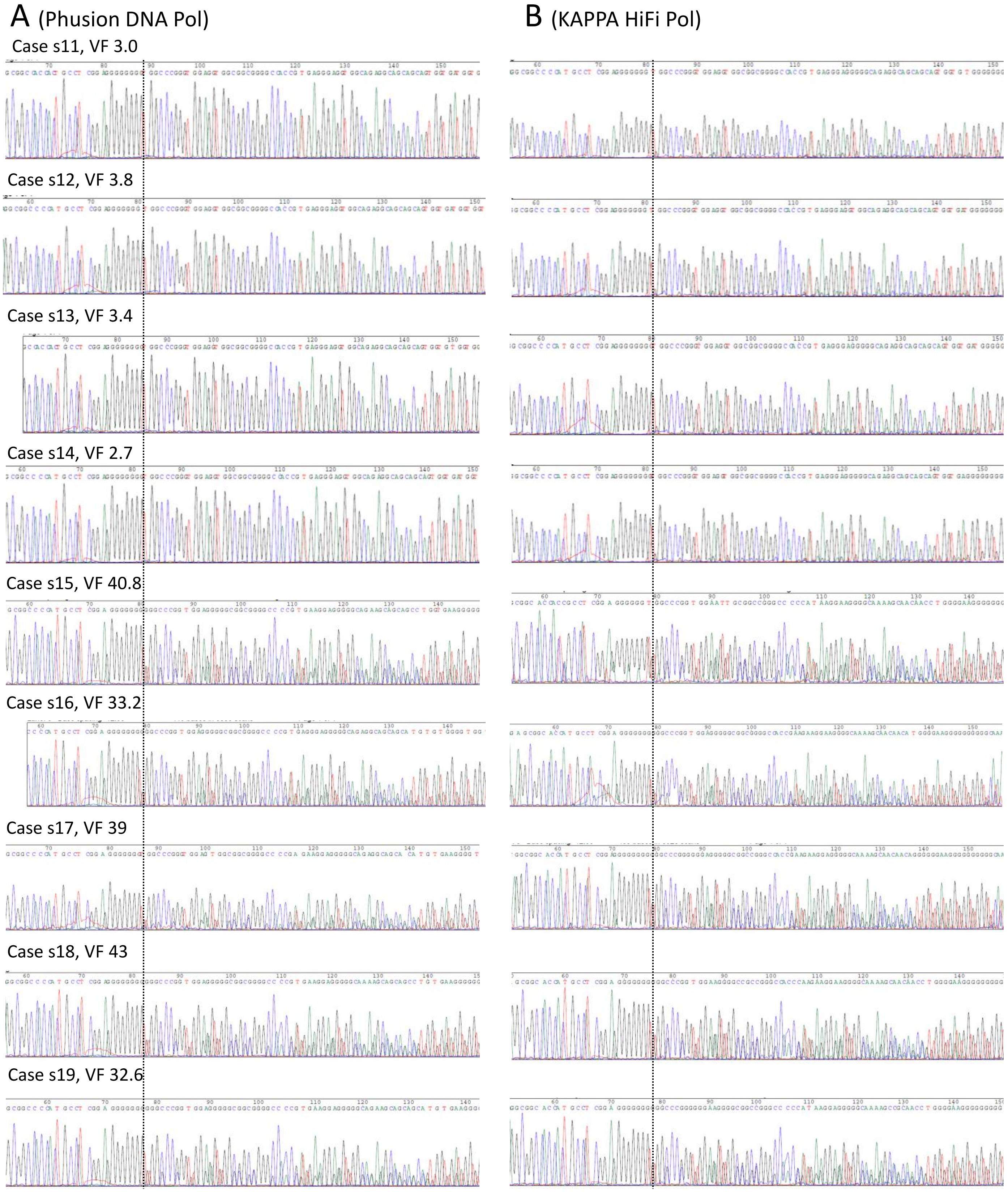
A (Phusion DNA Pol)
B (KAPPA HiFi Pol)
Case s11, VF 3.0
Case s12, VF 3.8
Case s13, VF 3.4
Case s14, VF 2.7
Case s15, VF 40.8
Case s16, VF 33.2
Case s17, VF 39
Case s18, VF 43
Case s19, VF 32.6
