# Supplementary material for: Clinical molecular testing for ASXL1 c.1934dupG p.Gly646fs mutation in hematologic neoplasms in the NGS era
Source: PLoS One. 2018 Sep 17;13(9):e0204218. doi: 10.1371/journal.pone.0204218 (PMC6141087; doi:10.1371/journal.pone.0204218)
Supplement: S2 Fig — Each column represents a single patient. Information regarding diagnosis, age, gender, outcome in the last follow-up, total number of mutations and specific mutations found is given. ASXL1 c.1934dupG was found co-mutated with TET2 (14 events), EZH2 (11 events), IDH2 (10 events), RUNX1 (10 events), NRAS (7 events) and DNMT3A (6 events). The number of events was <5 for IDH1, MLL/KMT2A, PTPN11, KRAS, FLT3, EGFR, JAK2, MPL, KIT, GATA2, TP53 and NOTCH1. DNMT3A, IDH1, IDH2 and RUNX1 mutations were frequent in MDS, MDS/MPN and AML NOS, with MDS related changes and AML secondary to MPN. FLT3 and NPM1 mutations were restricted to cases with AML, NOS and MDS/MPN (FLT3:c.2134A>T p.R712W, FLT3:c.2812_2820dupAATTTGACT p.N938_T940dupNLT, FLT3:c.2503G>T p.D835Y, NPM1: c.393_395dupGGA p.E131dup). All these mutations (DNMT3A, IDH1, IDH2, RUNX1, NPM1 and FLT3) were however absent in all 4 patients with MPN. MPN patient samples were characterized by the presence of JAK2 (c.1849G>T p.V617F), KIT (c.1655T>C p.M552T) and MPL mutations (MPL:c.1544G>T p.W515L and MPL:c.1841G>A p.G614E). Of these genes, other MPL mutations were found in one additional case with a diagnosis of AML NOS. (PDF) [file pone.0204218.s002.pdf]

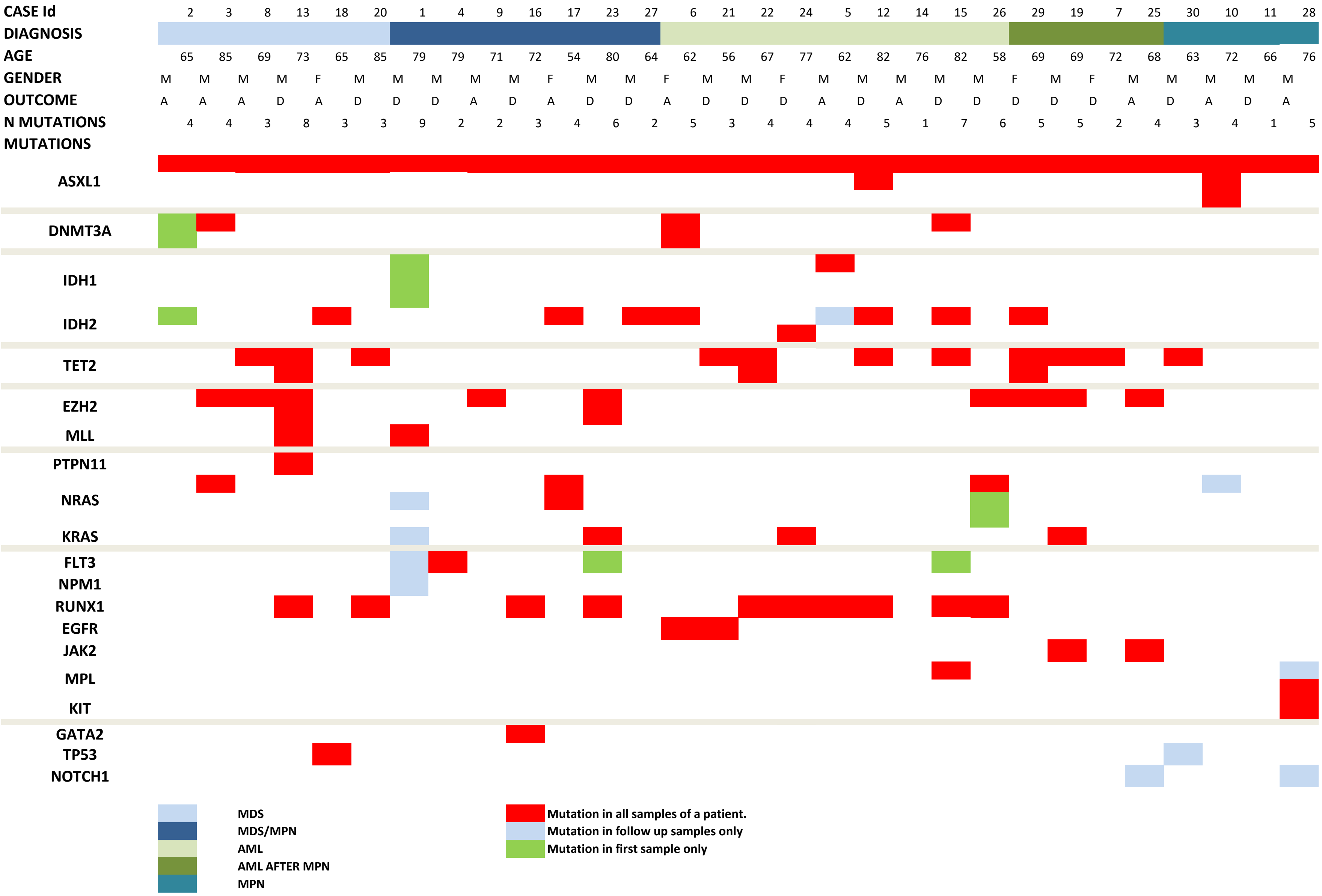
CASE Id
2 3 8 13 18 20 1 4 9 16 17 23 27 6 21 22 24 5 12 14 15 26 29 19 7 25 30 10 11 28
DIAGNOSIS
AGE
65 85 69 73 65 85 79 79 71 72 54 80 64 62 56 67 77 62 82 76 82 58 69 69 72 68 63 72 66 76
GENDER
M M M M F M M M M M F M M F M M F M M M M M F M F M M M M M
OUTCOME
A A A D A D D D A D A D D A D D D A D A D D D D D A D A D A
N MUTATIONS
4 4 3 8 3 3 9 2 2 3 4 6 2 5 3 4 4 4 5 1 7 6 5 5 2 4 3 4 1 5
MUTATIONS
ASXL1
DNMT3A
IDH1
IDH2
TET2
EZH2
MLL
PTPN11
NRAS
KRAS
FLT3
NPM1
RUNX1
EGFR
JAK2
MPL
KIT
GATA2
TP53
NOTCH1
MDS
MDS/MPN
AML
AML AFTER MPN
MPN
Mutation in all samples of a patient.
Mutation in follow up samples only
Mutation in first sample only
